# Corrosion inhibition of ductile iron in hydrochloric acid using 5-amino-1,3,4-thiadiazole-2-*thiol*: electrochemical and computational studies

**DOI:** 10.1038/s41598-026-51250-2

**Published:** 2026-05-12

**Authors:** Mohamed Helmy, Adham A. El-Zomrawy, Awad Sadek Mogoda, Ahmed Nasser, Mamdouh Mahmoud, Tarek A. Mohamed

**Affiliations:** 1 Chemistry Department, Faculty of Science, Al-Azhar University, Cairo, Egypt; 2 Chemistry Department, Faculty of Science, Cairo University, Giza, Egypt; 3 The Higher Institute of Engineering, New Elmarg, El-Qalyubia Egypt; 4 Faculty of Industry and Energy Technology, New Cairo Technological University, New Cairo, Egypt; 5 R&D Labs, Al-Nasr Castings Factory, Giza, Egypt

**Keywords:** Corrosion inhibition, Ductile iron, Electrochemical techniques, Adsorption isotherms, DFT, Monte Carlo simulation, Chemistry, Materials science

## Abstract

The corrosion inhibition performance of 5-amino-1,3,4-thiadiazole-2-thiol (5ATT) toward ductile iron in 1.0 M HCl solution was systematically investigated using complementary experimental and theoretical approaches, including weight loss measurements, potentiodynamic polarization, electrochemical impedance spectroscopy (EIS), and surface characterization (SEM/EDX), supported by density functional theory (DFT) calculations and Monte Carlo (MC) simulations. The results show that the inhibition efficiency increases markedly with inhibitor concentration, reaching ~ 81%, which indicates effective adsorption of 5ATT molecules on the ductile iron surface. Electrochemical measurements revealed a marked decrease in corrosion current density and a significant increase in charge transfer resistance, confirming the formation of a protective adsorbed film, which was further supported by surface analysis. Importantly, the present study provides a clear correlation between the molecular electronic properties and adsorption behavior of 5-ATT and its experimentally observed inhibition performance. Adsorption studies indicated strong interaction between the inhibitor molecules and the metal surface, while thermodynamic parameters suggested a mixed physisorption–chemisorption mechanism. Furthermore, theoretical calculations supported the experimental findings, demonstrating that both the electronic structure and adsorption configuration of 5-ATT play a key role in its corrosion inhibition efficiency. Therefore, 5-ATT exhibits high inhibition performance and strong adsorption capability, highlighting its potential as an effective corrosion inhibitor for ductile iron in acidic environments.

## Introduction

Ductile iron is widely utilized in the automotive, industrial, agricultural, and energy industries as well as infrastructure systems (such as water pipes, valves, and fittings) owing to its high strength, ductility, durability, and affordability. Using electrochemical methods, weight loss, pit depth measurement, surface morphology analysis, and corrosion product characterization, its corrosion performance in chloride-containing settings has been compared to that of carbon steel^1^. Moreover, because of its peculiar microstructure, which consists of graphite nodules embedded in a ferritic/pearlitic matrix, ductile iron has different corrosion characteristics than regular carbon steel, particularly in hostile chloride conditions. Ductile iron is more resistant to corrosion than mild steel in a variety of settings, such as near-neutral media, NaCl and NaOH solutions, and chloride-activated soils, according to long-term exposure experiments (up to three months). Additionally, electrochemical methods, cathodic protection evaluations, comprehensive surface analysis, and corrosion product analysis were used to systematically investigate the corrosion behavior of ductile iron in near-neutral conditions^2^. The accelerated corrosion of ductile iron has been primarily attributed to elevated salt concentrations in chloride-rich environments. In addition, recent long-term studies (up to 180 days) have highlighted the influence of microstructural features on the corrosion resistance in near-neutral and alkaline media, particularly NaOH and NaCl solutions supported by electrochemical measurements, surface characterization^3^. In parallel, the corrosion inhibition performance of 5-amino-1,3,4-thiadiazole-2-thiol (5-ATT) has been explored for various metallic systems. Moreover, the corrosion inhibition of ASTM A-890-1B steel in aqueous NaCl (3.5%) using 5-ATT “inhibitor” was studied at different temperatures by the electrochemical linear polarization resistance technique^4^ favoring a spontaneous and exothermic adsorption process. Nevertheless, the inhibitory effect of 5-ATT on ductile iron corrosion was assessed in a soil-analog medium, where the compound exhibited high inhibition efficiency even under highly aggressive conditions^5^.

Additionally, the inhibition behavior of 5-ATT on mild steel in 1.0 M HCl at 303 K has been studied practicing weight loss, electrochemical (AC and DC) studies and X-ray photoelectron spectroscopy (XPS), confirming its high efficiency in acidic media^6^. Its performance has also been evaluated for aluminum in acidic solutions through combined experimental and theoretical approaches, showing concentration-dependent efficiency that decreases at elevated temperatures^7^. The weight loss results indicated that the inhibition efficiency increased by rising the concentration of 5-ATT but lowered at elevated temperatures.

Recent studies have further confirmed the high efficiency of such organic inhibitors and their strong adsorption behavior on steel and iron surfaces in acidic media, highlighting their role in improving corrosion resistance and providing insight into structure–activity relationships^8–10^. Generally, effective acid corrosion inhibitors are organic compounds containing heteroatoms such as N, S, P, and/or O), along with π-electron systems (double bonds and aromatic rings), which enhance adsorption onto the metal surface^11–17^. Therefore, thiadiazole derivatives, often exhibit superior inhibition performance compared to those containing a single heteroatom^10,11^. While thiadiazole derivatives, including 5-amino-1,3,4-thiadiazole-2-thiol (5-ATT), which exists in thiol and thione tautomeric forms have been widely reported as effective corrosion inhibitors in acidic media^18–25^, their application to ductile iron has received very limited attention. This is especially crucial because of heterogeneous microstructure ductile iron, where micro-galvanic corrosion can be caused by graphite nodules imbedded in the metallic matrix, resulting in behavior that is very different from that of traditional steels. More significantly, despite 5-ATT has a well-known thiol–thione tautomerism, its impact on adsorption behavior and corrosion inhibition efficacy has not been thoroughly investigated, remarkably in a combined experimental–theoretical context. To the best of our knowledge, no prior research has used both experimental methods and molecular-level simulations to examine the corrosion inhibition behavior of 5-ATT on ductile iron in 1.0 M HCl while directly linking tautomeric structure with adsorption mechanism.

Therefore, the present work aims to bridge this gap by providing a comprehensive investigation of 5-ATT as a corrosion inhibitor for ductile iron, combining gravimetric, electrochemical, and surface characterization methods with density functional theory (DFT) calculations and Monte Carlo (MC) simulations. Particular emphasis is placed on elucidating the role of thiol–thione tautomerism in governing adsorption behavior and inhibition efficiency at both macroscopic and molecular scales.

## Material and methods

### Materials

**Scheme 1:**
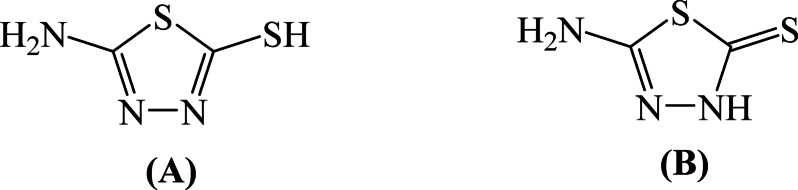
The chemical structure of: (**A**) 5-Amino-1,3,4-thiadiazole-2-*thiol*; (**B**) 5-Amino-1,3,4-thiadiazole-2(3H)-*thione*.

The experiments were performed using ductile iron specimen with compositions of: 93.4% Fe, 3.67% C, 2.340% Si, 0.004% S, 0.012% P, 0.190% Mn, 0.062% Ni, 0.097% Cr, 0.001% Mo, 0.018% V, 0.078% Cu, 0.009% Ti, 0.004% As, 0.010% Sn, 0.004% Al, 0.002% Pb, 0.004% Bi, 0.045% Mg and 0.006% Zn. The specimens were abraded with a series of emery papers from 400 to 2500 grit to achieve a mirror-like surface and washed thoroughly with double distilled water, de-greased with acetone and air dried. The solutions were prepared by the dilution of analytical grade 37% HCl with double distilled water in the absence and presence of 5-ATT as inhibitor in the concentration range from 0.05 to 5.0 mM. The molecular structure of 5-ATT), is shown in Scheme 1.

### Methods

#### Corrosion techniques

##### Weight loss measurements

Weight loss measurements were conducted in 100 mL of 1.0 M HCl (test solution), in the absence and presence of varying concentrations of 5-ATT, at 25 °C. Rectangular ductile iron specimens with dimensions of 1.0 × 2.0 × 0.5 cm^3^ (cut from a ductile iron specimen), accurately weighed, and immersed in the test solutions. The immersion period extended to 120 h, with mass measurements recorded at 12 h intervals. Corrosion rates were determined from the difference between the initial and final weights of the specimens. These experiments were performed to assess the inhibition efficiency of 5-ATT toward ductile iron in acidic medium. All weight loss measurements were performed in triplicate to ensure reproducibility, and the average values were reported.

##### Electrochemical measurements

Electrochemical experiments were carried out in a thermostatically controlled, water-jacketed glass cell with a capacity of 100 mL, employing a conventional three-electrode system. The setup consisted of a ductile iron working electrode, a platinum counter electrode, and an Ag/AgCl reference electrode. The working electrode was prepared as a square specimen (cut from ductile iron) with an exposed surface area of 1.0 × 1.0 cm^2^. All measurements were performed using a Gamry Reference 3000 potentiostat/galvanostat. Electrochemical measurements were conducted in naturally aerated 1.0 M HCl solution at 25 °C. Potentiodynamic polarization studies were performed, and the linear portions of the anodic and cathodic Tafel regions were extrapolated to the corrosion potential (E_corr_) to determine the corrosion current density (I_corr_). Polarization curves were recorded at a scan rate of 0.5 mV s^−1^^26^. Electrochemical impedance spectroscopy (EIS) measurements were carried out at the open circuit potential using a sinusoidal AC signal of 10 mV amplitude (peak-to-peak) over a frequency range from 100 kHz to 0.1 Hz. To guarantee reproducibility, every electrochemical measurement was performed at least three times. The Nyquist form of the impedance diagrams including representative data are offered since the produced curves showed great agreement with minimal fluctuation.

#### Surface analysis

The specimens were immersed in 100 mL of 1.0 M HCl solution, in the absence and presence of 5.0 mM 5-ATT, for 10 h. After immersion, the specimen samples were thoroughly rinsed with distilled water, dried, and subjected to surface morphology and compositional analyses using scanning electron microscopy (SEM Model Quanta 250 FEG, Field Emission Gun) attached with Energy Dispersive X-ray (EDX) Analyses, operated at an accelerating voltage of 30 kV. The microscope provides a magnification range from 14 × up to 1,000,000× with a resolution of approximately 1 nm.

#### Quantum chemical calculations

The DMol^3^ module^27,28^ in *BIOVIA* Materials Studio 6.0 (17.1.0.48) software^29^, is a potent density functional theory (DFT)-based quantum–mechanical modeling tool that is frequently used for investigations of electronic structure and surface interactions, such as corrosion inhibitor adsorption, molecular reactivity, and material characteristics. Thus, DMol^3^ has been used to evaluate the electronic properties of 5-ATT *thiol* and *thione* tautomers (Scheme 1) in gaseous and solvent environments. Geometry optimization and electronic structure calculations were performed with density functional theory approximations^30^ using the Generalized Gradient Approximation (GGA) with the BLYP (B88 exchange + LYP correlation) functionals^31,32^. A double numerical basis set is used herein with polarization functions (DNP 3.5), comparable in quality to the Gaussian 6-31G (d,p) basis set, was employed as implemented in the DMol^3^ module. Our computation favors the *thione* tautomer in the gaseous and solvent phases by 7.69 and 8.05 kcal/mole, respectively. Moreover, the energies of Frontier Molecular Orbitals (FMOs) of the Highest Occupied Molecular Orbital (E_HOMO_) and that of the Lowest Unoccupied Molecular Orbital (E_LOMO_) as well as electron density (ED) distributions were determined for the fully optimized structures. Solvent effects were incorporated to better simulate the corrosive medium. Monte Carlo (MC) simulations were subsequently carried out using the Adsorption Locator module within the same software package to investigate the adsorption behavior and preferred configuration of the inhibitor on the Fe (110) surface. The iron substrate was modeled as a five-layer slab cleaved along the (110) crystallographic plane and extended into a (15 × 15) supercell to minimize edge effects. The simulation cell dimensions were fixed at 24.8 × 24.8 × 38.1 Å. Adsorption simulations were performed in both gas and aqueous environments. To reproduce the acidic corrosive environment, the solvent system consisted of 100 H_2_O, 5 H_3_O⁺, and 10 Cl^−^ molecules. Each simulation was run for 100,000 steps to ensure adequate configurational sampling and proper equilibration of the system, following standard protocols widely adopted in corrosion inhibition modeling studies^18^.

## Results and discussion

### Weight loss measurements

#### Effect of contact time

**Fig. 1:**
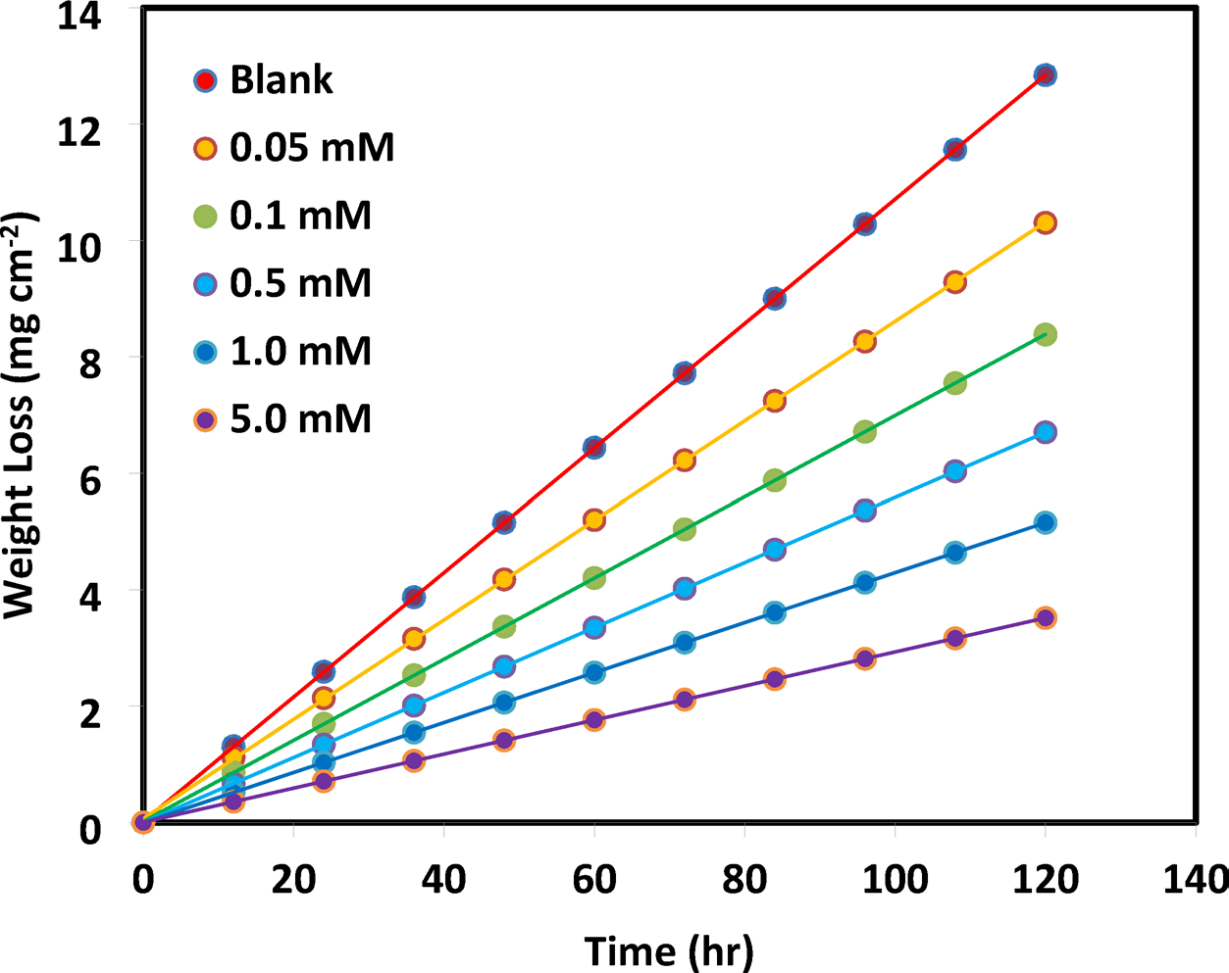
Ductile iron weight loss versus time curves in 1.0 M HCl solution containing different contaminants of 5-ATT at 25 °C.

The weight loss per area (mg cm^−2^) of Ductile Iron immersed in 1.0 M HCl solution at 25 °C for exposure periods up to 120 h. in the absence and presence of the inhibitor “5-ATT”, is displayed in Fig. 1. The corrosion rates (CR), surface coverage (θ), and inhibition efficiency (IE_w_) were calculated using Eqs. 1, 2, and 3, respectively, based on the weight loss data^33^.

1 $$CR\left( {{\mathrm{mpy}}} \right) = {534}W/Ad\;t$$

2 $$q = (CR - CR_{inh} )/CR$$

3 $$IW_{{\mathrm{w}}} \% = q \times {1}00$$

where *W* is the weight loss (mg) for Ductile Iron, *A* is the total surface area of the coupon (in^2^), *d* is density (g cm^3^), *t* is the immersion time (h). According to these results, increasing the inhibitor concentration leads to a marked reduction in the weight loss and corrosion rate of ductile iron. The linear increase in weight loss with immersion time suggests a constant corrosion rate, while the nearly parallel behavior observed in the presence of the inhibitor indicates stable inhibition efficiency throughout the exposure period. The adsorption of inhibitor molecules onto the ductile iron surface creates a protective coating that prevents aggressive H⁺ and Cl^−^ ions from accessing active corrosion sites, is responsible for the progressive decrease seen with increasing 5-ATT concentration. The increase in surface coverage (θ) with increasing inhibitor concentration indicates the progressive occupation of active sites on the metal surface, thereby reducing the effective metal–solution interfacial area available for corrosion reactions^34^, hence a pronounced decrease in the corrosion rate of ductile iron, refer to Table 1 for gravimetric results.

**Table 1:** Weight loss parameters and IE_i_% values for ductile iron corrosion in 1.0 M HCl solution in the absence and presence of various 5-ATT concentrations at 25 °C.

C_inh_ (mM)	weight loss rate (mg cm^−2^ h^−1^(	Corr. Rate (mpy)	θ	IE_w_%
0.00	0.107 ± 0.003	47.2 ± 1.2	…	…
0.05	0.085 ± 0.002	37.7 ± 0.9	0.201	20.1
0.10	0.070 ± 0.002	30.8 ± 0.8	0.348	34.8
0.50	0.056 ± 0.001	24.7 ± 0.7	0.478	47.8
1.00	0.043 ± 0.001	19.0 ± 0.5	0.598	59.8
5.00	0.029 ± 0.001	12.9 ± 0.4	0.726	72.6

In the absence of the inhibitor, ductile iron exhibits a high corrosion rate due to the aggressive attack of H⁺ and Cl^−^ ions at the metal–solution interface. The corrosion rate gradually decreases after 5-ATT is added, suggesting that the inhibitor molecules have effectively adsorbed onto the ductile iron surface and partially blocked the active corrosion sites. By limiting corrosive species’ access to the metal surface, this adsorption mechanism inhibits both cathodic hydrogen evolution processes and anodic metal dissolution. The corresponding increase in inhibition efficiency (IE_w_%) with increasing inhibitor concentration reflects a higher surface coverage (θ), indicating that a larger fraction of the ductile iron surface becomes protected as more inhibitor molecules are adsorbed^35,36^. At the highest concentration investigated (5.0 mM, 5-ATT inhibitor), the corrosion rate decreases by approximately 73%, confirming the strong inhibitive performance of 5-ATT under gravimetric conditions.

### Adsorption isotherms models

**Fig. 2:**
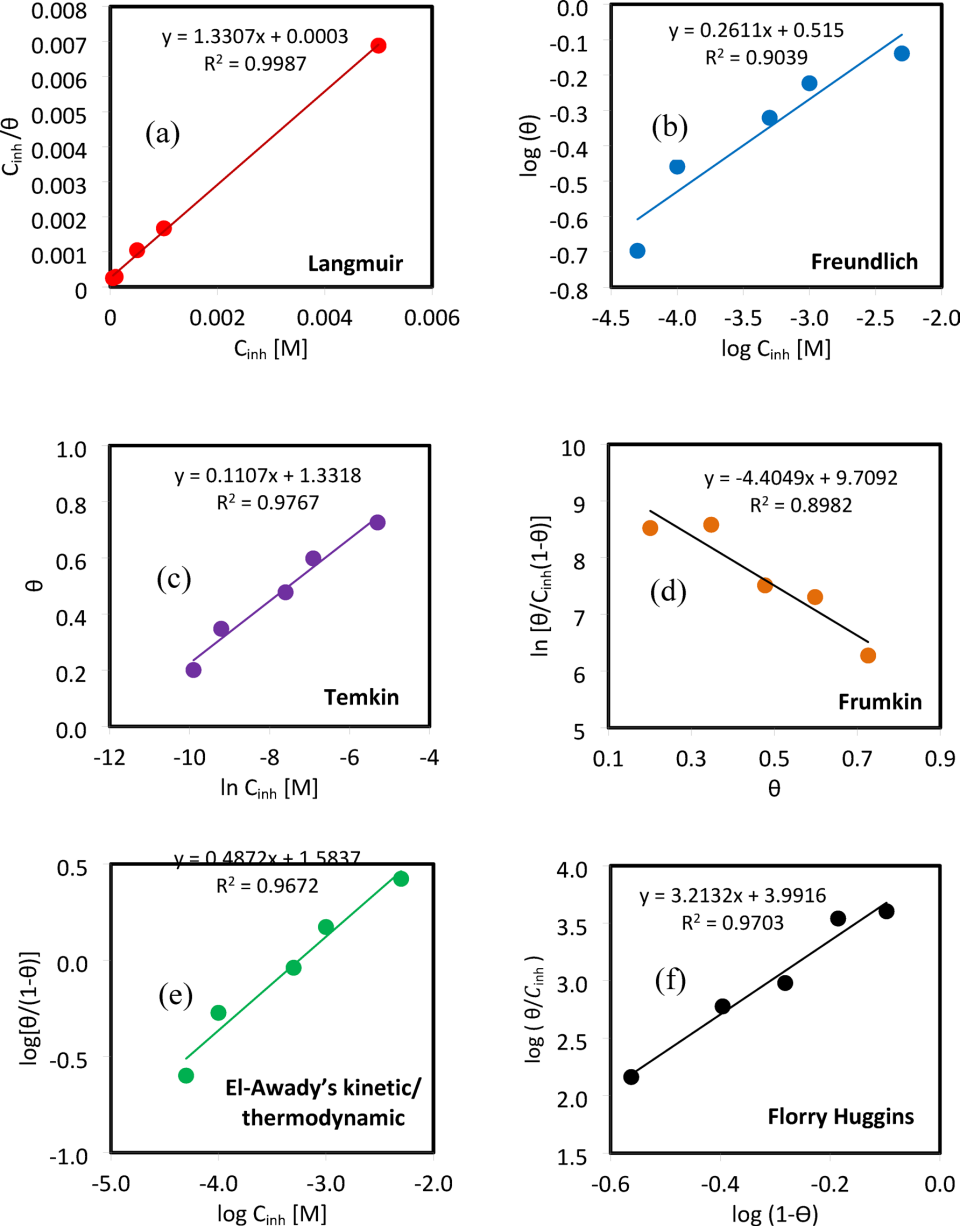
Linear fitting of the data of adsorption of 5-ATT to (**a**) Langmuir, (**b**) Freundlich, (**c**) Temkin, (**d**) Frumkin, (**e**) El-Awady and (**f**) Flory–Huggins isotherms for ductile iron in 1.0 M HCl solution at 25 °C.

Although numerous adsorption isotherms have been proposed to interpret experimental data^37,38^, not all are suitable for describing corrosion inhibitor adsorption at the metal–solution interface. Several widely used adsorption isotherm models, such as Langmuir, Freundlich, Temkin, Frumkin, Flory–Huggins, and the kinetic–thermodynamic (El-Awady), were used herein to examine the adsorption behavior and potential adsorption scenarios of 5-ATT on the ductile iron surface^39–45^. Since the inhibitor adsorption may entail surface heterogeneity, lateral interactions between adsorbed species, or multi-site adsorption mechanisms, these models were first investigated to investigate potential adsorption scenarios. The corresponding adsorption parameters are inferred from the slope, intercept, or both, obtained from plots of fractional surface coverage area (θ) versus inhibitor concentration (C_inh_) (Fig. 2a–f). The mathematical expressions of these isotherms are summarized in Table 2.

**Table 2:** Conventional and linear forms of the most used adsorption isotherms.

Isotherm model	Equations		References
	Non-linear form	Linear form	
Langmuir	*θ /(1 − θ)* = *K*_*ads*_* C*_*inh*_	*C*_*inh*_/*θ* = 1/*K*_*ads*_ + *C*_*inh*_	^33,39,40^
Freundlich	*θ* = *K*_*ads*_* (C*_*inh*_*)*^*1/n*^	*log θ* = *log K*_*ads*_ + *1/n log C*_*inh*_	^41^
Temkin	*exp(− 2aθ)* = *K*_*ads*_* C*_*inh*_	*θ* = *− 1/2a ln K*_*ads*_* − 1/2a ln C*_*inh*_	^42^
Frumkin	*[θ /(1 − θ)] exp(− 2αθ )* = *K*_*ads*_* C*_*inh*_	*ln [θ / C*_*inh*_* (1 − θ)]* = *ln K*_*ads*_ + *2αθ*	^43^
Flory–Huggins	*θ /χ(1 − θ)*^*χ*^ = *K*_*ads*_* C*_*inh*_	*log (θ / C*_*inh*_*)* = *log (χ K*_*ads*_*)* + *χ log (1 − θ)*	^41,44,45^
El-Awady	*[θ /(1 − θ)]*^*1/y*^ = *K C*_*inh*_	*log [θ /(1 − θ)]* = *log K* + *y log C*_*inh*_	^43^

Although the Langmuir isotherm is widely applied for corrosion inhibitor adsorption on metal surfaces, and showed a high correlation coefficient (R^2^ = 0.9987) in the present work, the deviation of the slope from unity indicates that ideal monolayer adsorption on a homogeneous surface is not fully satisfied^46,47^. This deviation suggests the presence of surface heterogeneity, intermolecular interactions between adsorbed species, and/or multi-site adsorption behavior. Similarly, the Freundlich (R^2^ = 0.9039) and Frumkin (R^2^ = 0.8982) models showed relatively poorer agreement with the experimental data and are therefore considered less representative for the present system^41,45,48–50^.

Greater emphasis is placed on the Temkin, El-Awady, and Flory–Huggins isotherms, which provide better agreement with the experimental data, with correlation coefficients of 0.9767, 0.9672, and 0.9703, respectively (Table 3). The Temkin model reflects adsorbate–adsorbate interactions on a heterogeneous surface^42^, while the kinetic–thermodynamic (El-Awady) model provides a more realistic description of the adsorption process by allowing multi-site adsorption, where one inhibitor molecule may occupy more than one active site. The obtained value of 1/y > 1 suggests that the inhibitor molecules adsorb through multiple adsorption centers, supporting the proposed mechanism^43,51^. The Flory–Huggins model further supports the multi-site adsorption mechanism, suggesting that each inhibitor molecule displaces several adsorbed water molecules from the metal surface^52^. Therefore, the non-ideal adsorption models that take surface heterogeneity, intermolecular interactions, and multi-site adsorption into consideration better characterize the adsorption of 5-ATT “inhibitor” on ductile iron surface. Both the correlation coefficient (R^2^) and the consistency of the acquired parameters with the physicochemical properties of the adsorption process were taken into consideration while choosing the best adsorption isotherm.

**Table 3:** Adsorption isotherms parameters of 5-ATT as a corrosion inhibitor of ductile iron in 1.0 M HCl.

Isotherm model	Isotherm parameters			
	Slope	K_ads_	Linear correlation (R^2^)	ΔG°_ads_ (kJ mol^−1^)
Langmuir	1.331	3333	0.9987	− 30.0
Freundlich	0.261	0.515	0.9039	− 8.3
Temkin	0.111	167,831	0.9767	− 39.8
Frumkin	− 4.405	16,468	0.8982	− 34.0
Flory–Huggins	3.213	3053	0.9703	− 29.8
El-Awady	0.487	1781	0.9672	− 28.5

### Standard Gibbs free energy

Thermodynamic functions that clarify the nature of the inhibitor–metal interaction can be computed using the adsorption parameters obtained from the isotherm models. When assessing the thermodynamics of inhibitor adsorption on metal surfaces in corrosion systems, the standard Gibbs free energy of adsorption (ΔG°_ads_) is crucial. The corrosion inhibition performance is directly influenced by the spontaneity, strength, and kind of adsorption process, all of which are revealed by the value of ΔG°_ads_. In general, a negative ∆G°_ads_ value indicates a spontaneous adsorption process, whereas a positive value suggests non-spontaneous adsorption^34,53^.

The magnitude of ΔG^°^_ads_ provide insight into the nature of the adsorption mechanism. Values around − 20 kJ mol^−1^ or less negative are generally associated with physisorption, which involves electrostatic interactions between the charged metal surface and the inhibitor molecules. In contrast, values of − 40 kJ mol^−1^ or more negative typically indicate chemisorption, involving the formation of coordination bonds through charge sharing or electron transfer between the inhibitor “5-ATT hetero atoms” and the metal surface^54–57^. The adsorption equilibrium constant (K_ads_) obtained from the isotherm analysis is related to the standard Gibbs free energy (ΔG^°^_ads_) of adsorption through the thermodynamic relationship^58^ described in Eq. (4) which has been rearranged in Eq. (5) where R is the universal gas constant, T is the absolute temperature, and 55.5 represents the molar concentration of water in solution (mol L^−1^).

4 $$K_{{{\mathrm{ads}}}} = \frac{1}{{C_{{H_{2} O}} }}{\mathrm{exp}}( - \, \Delta G^{^\circ }_{{{\mathrm{ads}}}} /{\mathrm{RT}})$$

5 $${\Delta }G_{ads}^{ \circ } = - RT\;{\mathrm{ln}}\left( {55.5\,K_{ads} } \right)$$

Table 3 summarizes the computed ΔG°_ads_ values, and the values change depending on the adsorption isotherm used. This diversity results from the many underlying assumptions of each model, especially in relation to adsorption site distribution, intermolecular interactions, and surface homogeneity. Since these models were found to provide a more physically realistic description of the adsorption process, the ΔG°_ads_ values produced from the Temkin, kinetic–thermodynamic (El-Awady), and Flory–Huggins isotherms are given higher weight in the current study (Section “Adsorption isotherms models”). These models account for surface heterogeneity, lateral interactions between adsorbed species, and multi-site adsorption behavior, which are consistent with the characteristics of ductile iron surfaces. Although the Langmuir isotherm exhibited a high correlation coefficient, the deviation of its slope from unity indicates that ideal monolayer adsorption is not strictly followed^46–48^. Therefore, the ΔG^°^_ads_ value derived from the Langmuir model is considered less reliable for mechanistic interpretation. The obtained ΔG^°^_ads_ values (Table 3) are negative, indicating that the adsorption process is spontaneous. Furthermore, the magnitude of ΔG^°^_ads_ suggests that the adsorption of 5-ATT on ductile iron involves a mixed physisorption–chemisorption mechanism, which is consistent with the adsorption behavior discussed above and supported by the experimental and theoretical results.

### Potentiodynamic polarization data measurements

**Fig. 3:**
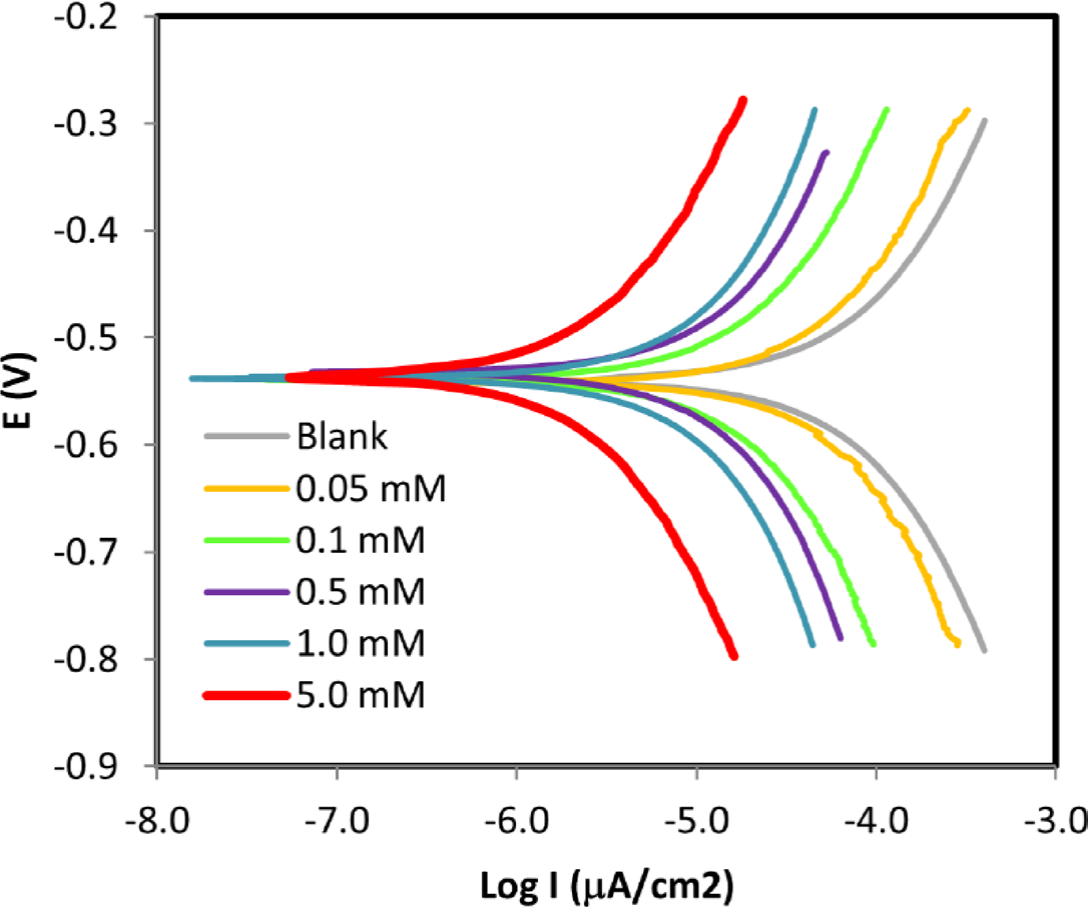
Potentiodynamic polarization curves for ductile iron in 1.0 M HCl as a function of 5-ATT concentration at 25 °C.

Figure 3 illustrates the potentiodynamic polarization curves of ductile iron in 1.0 M HCl solution in the absence and presence of various concentrations of 5-ATT at 25 °C. The corresponding electrochemical parameters-namely, the corrosion potential (E_*corr*_), corrosion current density (i_*corr*_), anodic (β_*a*_) and cathodic (β_*c*_) Tafel slopes, corrosion rate, and inhibition efficiency (IE_i_%), were calculated using Eq. (6) ^58–61^, the results are summarized in Table 4.

6 $${\mathrm{IE}}_{{\mathrm{i}}} {{\% }} = {\mathrm{ln}}\left[ {\frac{{({\mathrm{i}}_{corr}^{^\circ } - {\mathrm{i}}_{corr} )}}{{{\mathrm{i}}_{corr}^{^\circ } }}} \right] \times 100$$

**Table 4:** Polarization parameters and IE_i_% values for ductile iron corrosion in 1.0 M HCl solution in the absence and presence of various 5-ATT concentrations at 25 °C.

C_inh_ (mM)	− E_corr_ (mV)	i_corr_ (µA cm^−2^)	β_a_ (V dec^−1^)	β_c_ (V dec^−1^)	Corr. Rate (mpy)	Chi Squared χ^2^	IE_i_ (%)
0.00	540	94.8	0.348	0.367	43.99	0.01	…
0.05	541	46.0	0.300	0.330	21.3	0.02	51.5
0.10	545	34.8	0.240	0.260	16.1	0.01	63.3
0.50	539	29.5	0.190	0.210	13.7	0.02	68.9
1.00	537	23.4	0.165	0.175	10.8	0.01	75.3
5.00	544	17.6	0.140	0.150	8.2	0.01	81.4

The corrosion current density (i_*corr*_) decreases markedly with increasing inhibitor concentration. Specifically, i_*corr*_ decreases from 94.8 µA cm^−2^ in the uninhibited solution to 17.6 µA cm^−2^ at 5.0 mM 5-ATT, corresponding to an inhibition efficiency of approximately 81%. This pronounced reduction in i_*corr*_ demonstrates the effective suppression of the corrosion kinetics of ductile iron in acidic medium. In contrast, the anodic (β_*a*_) and cathodic (β_*c*_) Tafel slopes exhibit only slight and gradual variations upon addition of the inhibitor, suggesting that the fundamental mechanisms of anodic metal dissolution and cathodic hydrogen evolution remain essentially unaffected. According to the widely accepted criterion, if the displacement in corrosion potential (E_corr_) is less than ± 85 mV, the inhibitor is classified as mixed-type. In the present study, the observed shift falls within this range, indicating that 5-ATT acts as a mixed-type inhibitor, influencing both anodic metal dissolution and cathodic hydrogen evolution reactions. This inhibitory behavior is further evidenced by the corrosion rate values (expressed in mpy), which decrease from 43.99 to 8.2 mpy upon increasing the inhibitor concentration to 5.0 mM. Moreover, the low chi-squared (χ^2^) values obtained from the fitting procedure validate the reliability and accuracy of the polarization data analysis. Over-all, the inhibitor suppresses both anodic and cathodic reactions predominantly through surface adsorption and the formation of a protective film that retards charge transfer processes at the metal-solution interface, in good agreement with the gravimetric results^62,63^.

### Electrochemical impedance spectroscopy (EIS) measurements

**Fig. 4:**
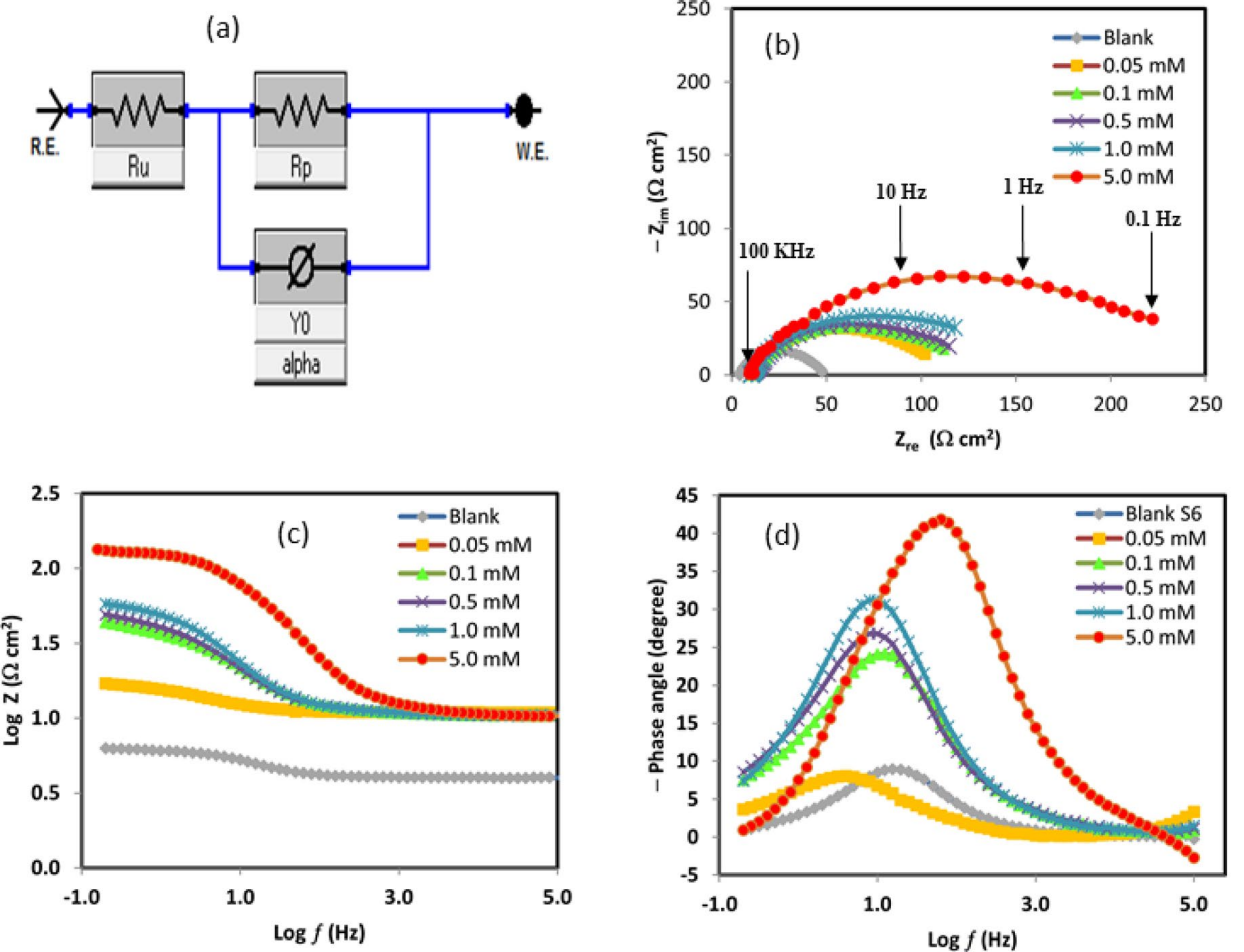
EIS, (**a**) Equivalent circuit, (**b**) Nyquist, (**c**) Bode Impedance, and (**d**) Bode phase angle plots of ductile iron in 1.0 M HCl as a function of 5-ATT concentration at 25 °C.

EIS was employed to further elucidate the corrosion inhibition performance of 5-ATT for ductile iron in 1.0 M HCl solution. The Nyquist plots obtained at 25 °C with and without different inhibitor doses are displayed in Fig. 4b. The impedance spectra consistently show a single depressed capacitive semicircle (loop), suggesting that charge transfer at the metal-solution interface primarily controls the corrosion process. To account for surface heterogeneity and non-ideal capacitive behavior, an equivalent circuit consisting of the solution resistance (R_*u*_), charge transfer resistance (R*p*), and a constant phase element (CPE) was used to extract and evaluate the experimental impedance data.

The heterogeneous microstructure of ductile iron should be carefully considered, even if the impedance measurements show a single depressed capacitive semicircle, indicating that charge transfer is primarily responsible for the corrosion process. Graphite nodules embedded in a metallic matrix make up ductile iron, which can result in micro-galvanic interactions and other interfacial phenomena such inhibitor species adsorption and desorption. In principle, such processes may result in multiple time constants in the impedance response. However, the absence of clearly resolved additional loops in the Nyquist plots, together with the presence of only one broad phase maximum in the Bode plots, indicates that these processes are either overlapping within the same frequency domain or are not sufficiently separated to be individually distinguished under the present experimental conditions. Therefore, the use of a simplified equivalent circuit (Ru–Rp–CPE) is considered an adequate and physically meaningful approximation. This is further supported by the good agreement between the experimental and fitted data, as well as the low chi-squared (χ^2^) values obtained from the fitting procedure, confirming the reliability of the selected model. The deviation from ideal capacitive behavior, described by the CPE, arises from surface heterogeneity and the spread of relaxation times linked to the microstructure of ductile iron. In addition, the adsorption of 5-ATT molecules likely suppresses micro-galvanic activity by forming a protective layer over both graphite and metallic phases, thereby promoting a more homogeneous interfacial response. As shown in Table 5, the charge transfer resistance (R_*p*_) increases significantly with increasing inhibitor concentration, rising from 41.91 Ω·cm^2^ in the uninhibited solution to 218.20 Ω cm^2^ at 5.0 mM 5-ATT. This substantial increase in R_*p*_ indicates the formation of a protective adsorbed film that effectively impedes charge transfer processes at the ductile iron-solution interface^63–66^. In contrast, the solution resistance (R_*u*_) remains nearly constant upon addition of the inhibitor, indicating that the observed inhibition effect primarily arises from interfacial processes/phenomena rather than variations in electrolyte conductivity. The CPE magnitude (Y_o_) decreases markedly with increasing inhibitor concentration, accompanied by a decrease in the dispersion parameter (α) from 0.844 to 0.710. This behavior suggests enhanced surface coverage and increased surface heterogeneity due to the adsorption of 5-ATT molecules. The reduction in α reflects greater deviation from ideal capacitive behavior, likely resulting from a more heterogeneous and non-uniform charge distribution at the metal–solution interface.

**Table 5:** EIS parameters and IE_R_% values for ductile iron corrosion in 1.0 M HCl solution in the absence and presence of various 5-ATT concentrations at 25 °C.

C_inh_ (mM)	R_u_ (Ω·cm^2^)	R_p_ (Ω·cm^2^)	Y_0_ × 10^4^ (Ω^−1^ cm^−2^ sⁿ)	α	C_dl_ × 10^4^ (F cm^−2^)	IE_R_%
0.00	4.11	41.91	12.07	0.844	6.95	…
0.05	10.64	93.46	11.65	0.758	5.74	55.16
0.10	10.47	113.40	9.37	0.755	4.53	63.04
0.50	10.42	119.00	7.27	0.713	2.71	64.78
1.00	10.29	152.70	6.12	0.715	2.38	72.55
5.00	10.90	218.20	3.90	0.710	1.43	80.79

Although apparent double-layer capacitance (C_*dl*_) values were estimated for comparative purposes, the observed decrease in C_*dl*_ from 6.95 × 10^−4^ to 1.43 × 10^−4^ F cm^−2^ is primarily attributed to the replacement of adsorbed water molecules by inhibitor species and the consequent increase in the thickness of the protective adsorbed layer. This effect leads to a reduction in the local dielectric constant at the metal–solution interface^67,68^. A concentration-dependent enhancement in the impedance response is further evident, where the progressive increase in charge transfer resistance with 5-ATT concentration reflects the gradual strengthening of the protective adsorbed layer at the metal/solution interface. This trend is consistent with the surface protection behavior observed in morphological analysis^69–71^. The inhibition efficiency based on EIS measurements (IE_*R*_) was calculated using Eq. (7) ^63,72,73^:

7 $${\mathrm{IE}}_{R} {{\% }} = \frac{{{\mathrm{R}}_{p} - {\mathrm{R}}_{p}^{^\circ } }}{{{\mathrm{R}}_{p} }} \times 100$$

The (IE_*R*_%) values increase with inhibitor concentration, reaching 80.79% at 5.0 mM, in close agreement with those obtained from potentiodynamic polarization and gravimetric measurements. This strong consistency among different techniques confirms the reliability of the experimental results and further supports the proposed inhibition mechanism based on surface adsorption and protective film formation.

### Morphological analysis

#### Scanning electron microscope (SEM)

**Fig. 5:**
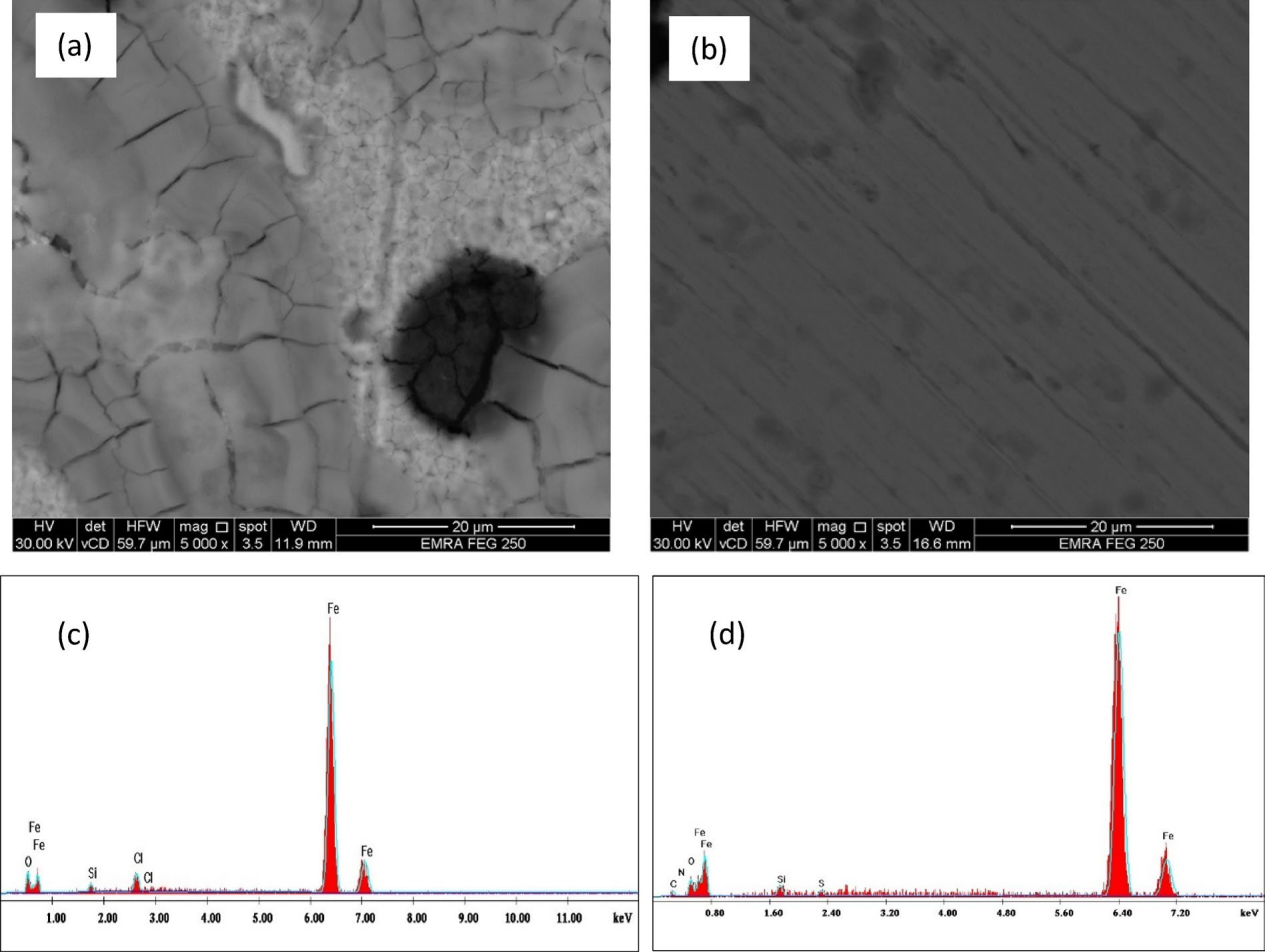
Scanning electron micrographs (SEM) of (**a**) Blank, (**b**) 5-ATT, and EDX spectra of (**c**) Blank, (**d**) 5-ATT, for ductile iron surface.

SEM was employed to examine the surface morphology of ductile iron specimens after immersion for 10 h in 1.0 M HCl solution in the absence and presence of 5.0 mM 5-ATT. The SEM micrograph of the specimen exposed to the uninhibited acidic solution (Fig. 5a) reveals pronounced surface degradation characterized by increased roughness, cracks, and localized corrosion features, indicating aggressive attack of HCl medium^74,75^.

In contrast, the SEM image of specimen immersed in the inhibited solution containing 5.0 mM 5-ATT (Fig. 5b) displays a considerably smoother and more homogeneous surface morphology with a marked reduction in visible corrosion defects at the examined magnification^76^. The improved surface integrity indicates that the inhibitor effectively mitigates corrosive attack through the formation of a protective adsorbed layer on the metal surface. These observations corroborate the electrochemical and gravimetric findings confirming that the adsorption of 5-ATT molecules leads to the formation of a barrier film that restricts/hinders the interaction between the corrosive medium and the ductile iron surface, thereby significantly reducing corrosion damage^77^.

#### Energy dispersive X-ray (EDX)

EDX analysis was performed to determine the elemental composition of the ductile iron surface before and after immersion in the inhibitor-containing solution. The EDX spectrum of the uninhibited specimen (Fig. 5c) mainly shows iron along with oxygen and minor silicon signals, confirming the formation of corrosion products and surface oxidation during exposure to the acidic medium^78^. In contrast, the spectrum obtained in the presence of 5.0 mM 5-ATT (Fig. 5d), reveals additional peaks corresponding to carbon, nitrogen, and sulfur, which are characteristic elements of the inhibitor molecule^79^. The detection of these elements provides strong evidence for the adsorption of 5-ATT species onto the ductile iron surface The formation of this inhibitor-derived surface layer is believed to act as a protective physical barrier that restricts the diffusion of aggressive species (such as H⁺ and Cl^−^ ions) toward the metal surface, thereby retarding both anodic metal dissolution and cathodic hydrogen evolution^80^. These observations are consistent with the electrochemical (polarization and EIS) and gravimetric results, further confirming the protective efficacy of 5-ATT against ductile iron corrosion in acidic media.

### Computational approach

#### Density functional theory (DFT) calculations

**Fig. 6:**
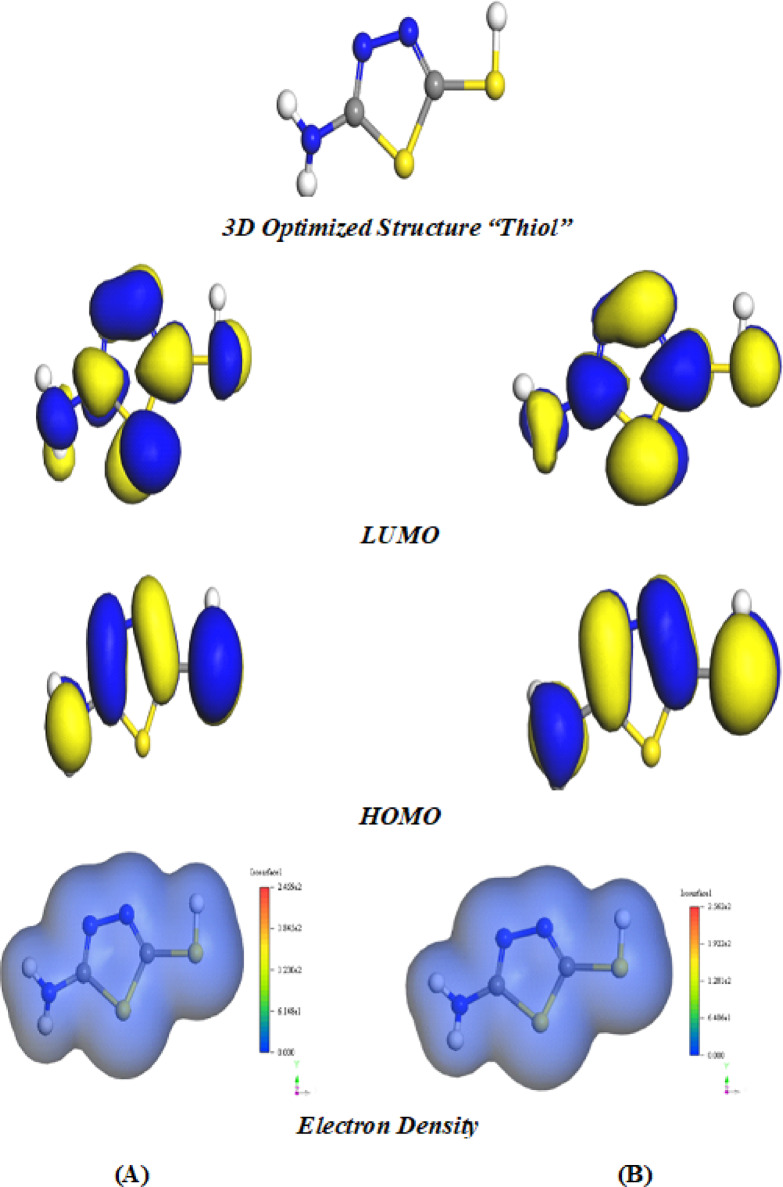
DFT Optimized structures, LUMO, HOMO and electron density (ED) of 5-ATT *thiol* inhibitor: (**A**) gas phase; (**B**) solvent phase.

**Fig. 7:**
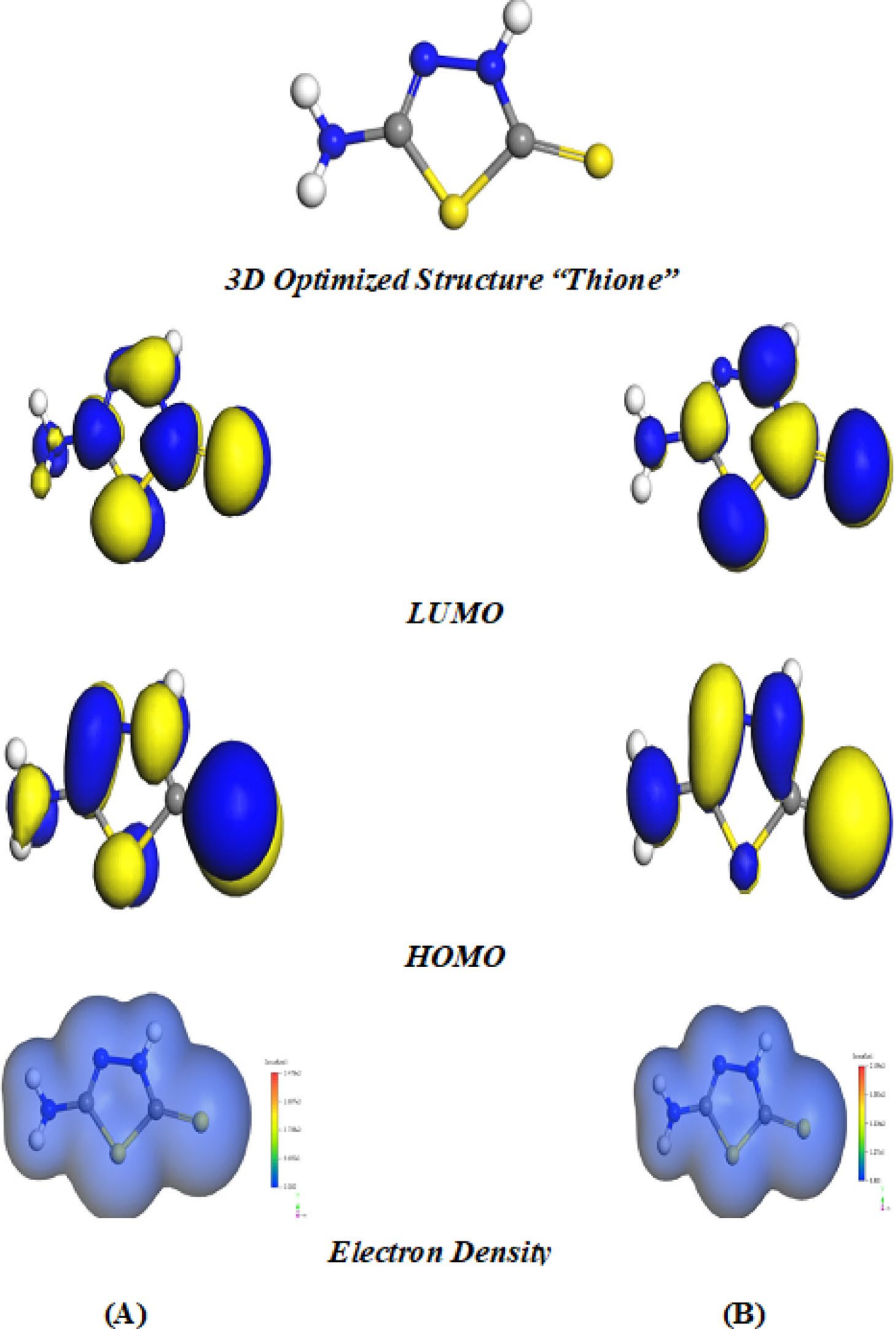
DFT Optimized structures, LUMO, HOMO and electron density (ED) of 5-ATT *thione* inhibitor: (**A**) gas phase; (**B**) solvent phase.

The electronic structure and corrosion inhibition behavior of the investigated 5-ATT inhibitor considering both *thiol* and *thione* tautomers (Scheme 1) were analyzed using DFT/BLYP in both gaseous and solvent phases. The optimized three-dimensional (3D) geometries, frontier molecular orbitals (FMOs), and electron density distribution corresponding to the lowest-energy configuration are presented in Fig. 6 (*thiol* form) and Fig. 7 (*thione* form), respectively. The optimized structures of both tautomers exhibit an extended quasi-planar conformation, which is favorable for adsorption on metallic surfaces as it promotes enhanced surface contact and facilitates effective orbital overlap with the metal surface^81,82^. Despite, despite their structural similarity, pronounced electronic differences arise because of the *thione-thiol* tautomeric transformation. As shown in Fig. 7, the HOMO of the *thione* form is predominantly localized over the sulfur atom of the C = S group and the adjacent nitrogen atoms of the thiadiazol ring, identifying these heteroatoms as the principal electron-donating centers. The calculated HOMO energies of − 4.666 eV (gas phase) and − 4.875 eV (solvent phase) suggest a strong propensity of the *thione* tautomer to donate electrons to the vacant 3d (Fe) orbitals^83,84^, refer to Table 6.

**Table 6:** Quantum chemical parameters of 5-ATT inhibitor (*Thione* and *Thiol*) calculated using density functional theory in gas and solvent phases.

	Inh	Phase	E(HOMO) (eV)	E(LUMO) (eV)	ΔE (eV)	A (eV)	I (eV)	X (eV)	η (eV)	σ (eV^−1^)	ΔN
BLYP	Thione	Gas	− 4.666	− 1.481	3.185	1.481	4.666	3.074	1.593	0.628	1.233
		Solvent	− 4.875	− 1.699	3.177	1.699	4.875	3.287	1.588	0.630	1.169
	Thiol	Gas	− 5.116	− 1.440	3.676	1.440	5.116	3.278	1.838	0.544	1.013
		Solvent	− 5.179	− 1.513	3.666	1.513	5.179	3.346	1.833	0.546	0.997
B3LYP	Thione	Gas	− 5.580	− 1.087	4.491	1.087	5.580	3.333	2.245	0.445	0.816
		Solvent	− 5.815	− 1.087	4.728	1.087	5.815	3.451	2.364	0.423	0.75
	Thiol	Gas	− 6.077	− 0.737	5.340	0.737	6.077	3.407	2.670	0.375	0.673
		Solvent	− 6.190	− 0.970	5.219	0.970	6.190	3.580	2.609	0.383	0.655

In comparison, the thiol tautomer (Fig. 6) exhibits slightly lower HOMO energies of − 5.116 eV (gas) and − 5.179 eV (solvent), indicating a comparatively reduced intrinsic electron-donating ability from a purely electronic perspective. Nevertheless, the HOMO density in thiol is highly concentrated around the sulfur atom of the –SH group and neighboring nitrogen atoms, implying that the –SH functionality introduces an additional localized adsorption center capable of coordinating with the iron surface. Conversely, the LUMO distributions in both tautomers are delocalized over π-conjugated framework and heteroatoms, facilitating electron acceptance from the metal surface through back-donation. The LUMO energies for *thione* (− 1.481 eV gas; − 1.699 eV solvent) are slightly lower than those of *thiol* (− 1.440 eV gas; − 1.513 eV solvent), indicating that *thione* has a marginally greater tendency of the *thione* tautomer to accept electron density from iron^85^. According to Koopmans’ theorem, the frontier orbital energies can be used to evaluate key global reactivity descriptors, including electron affinity (A), electronegativity (χ), ionization potential (I), and global hardness (η), which were calculated using Eqs. (8–11) ^29^:

8 $$A = - E_{{{\mathrm{LUMO}}}}$$

9 $$\chi = \frac{{ - \left( {E_{{{\mathrm{HOMO}}}} + E_{{{\mathrm{LUMO}}}} } \right)}}{2}$$

10 $$I = - E_{{{\mathrm{HOMO}}}}$$

11 $$\eta = \frac{{\Delta E_{{{\mathrm{gap}}}} }}{2}$$

The calculated global reactivity descriptors are summarized in Table 6. The energy gap (ΔE) of the *thione* tautomer is 3.185 eV (gas phase) and 3.177 eV (solvent phase), whereas the thiol tautomer exhibits larger gaps of 3.676 eV (gas) and 3.666 eV (solvent). The smaller ΔE values of *thione* tautomer indicate higher molecular reactivity and greater propensity for charge transfer, while the larger gap of thiol reflects comparatively greater electronic stability. Consistently, the global hardness (η) values of *thione* tautomer (1.593 eV gas; 1.588 eV solvent) are lower than those of thiol form (1.838 eV gas; 1.833 eV solvent), confirming the relatively softer character of the *thione* structure. Correspondingly, the higher softness (σ) and larger fraction of electron transfer (ΔN = 1.233 gas; 1.169 solvent) for *thione* tautomer compared lower softness of the thiol form (ΔN = 1.013 gas; 0.997 solvent) further support its stronger tendency to donate electrons to the Fe surface^86,87^.

The electron density (ED) maps in Fig. 6 and Fig. 7 corroborate these quantitative results revealing pronounced charge accumulation around sulfur and nitrogen atoms in both tautomers. However, the electron density in the *thione* form appears more delocalized over the C=S moiety, which enhances its π-electron interaction capability with the metal surface. Therefore, based on DFT-derived electronic descriptors, the *thione* tautomer exhibits greater intrinsic reactivity and a higher theoretical propensity for charge transfer toward the iron surface under isolated molecular conditions, prior to explicit environmental effects^88,89^.

To further assess the robustness of the results obtained, complementary calculations were carried out using the hybrid B3LYP functional (see Table 6). Although the absolute values of the computed quantum chemical parameters differ from those obtained using BLYP functionals, where the trend remains consistent. In both gas and solvent phases, the thione tautomer displays a smaller energy gap (ΔE) and reduced global hardness (η) relative to the thiol form, suggesting greater intrinsic reactivity and an enhanced tendency for charge transfer. The increase in values of ΔE out of B3LYP results can be ascribed to the incorporation of exact exchange in hybrid functionals, which typically leads to larger HOMO–LUMO energy gaps. Nevertheless, this systematic shift does not alter the relative ordering or the overall interpretation of the electronic properties. The consistency between the GGA (BLYP) and hybrid (B3LYP) approaches thus supports the robustness and reliability of the theoretical conclusions, particularly regarding the enhanced intrinsic reactivity of the *thione* tautomer.

#### Monte Carlo Simulation (MCs)

**Fig. 8:**
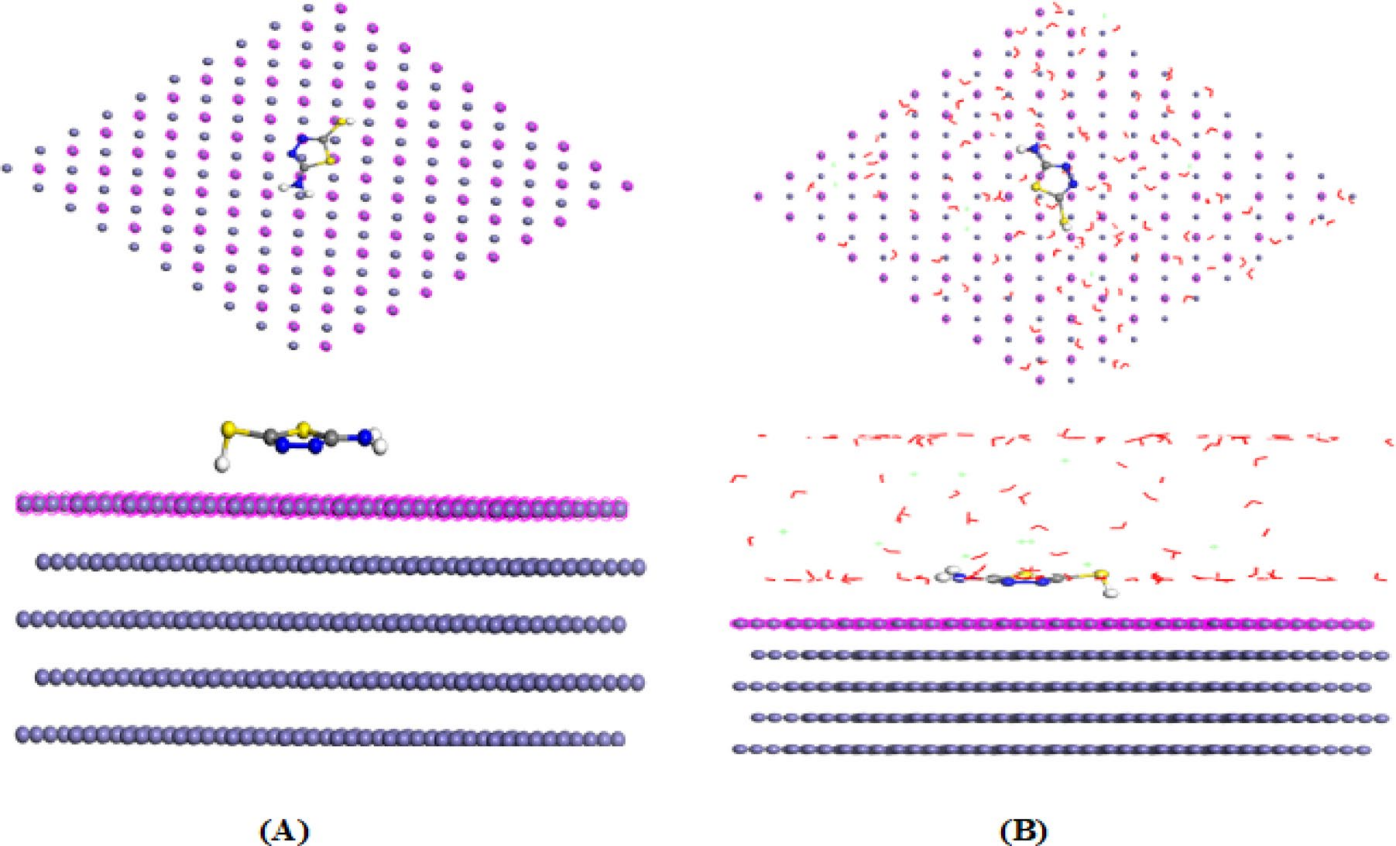
Equilibrium adsorption configuration of 5-ATT inhibitor, *thiol* adsorbed on the Fe (110) calculated by MC simulation at room temperature: (**A**) gas phase; (**B**) solvent phase.

**Fig. 9:**
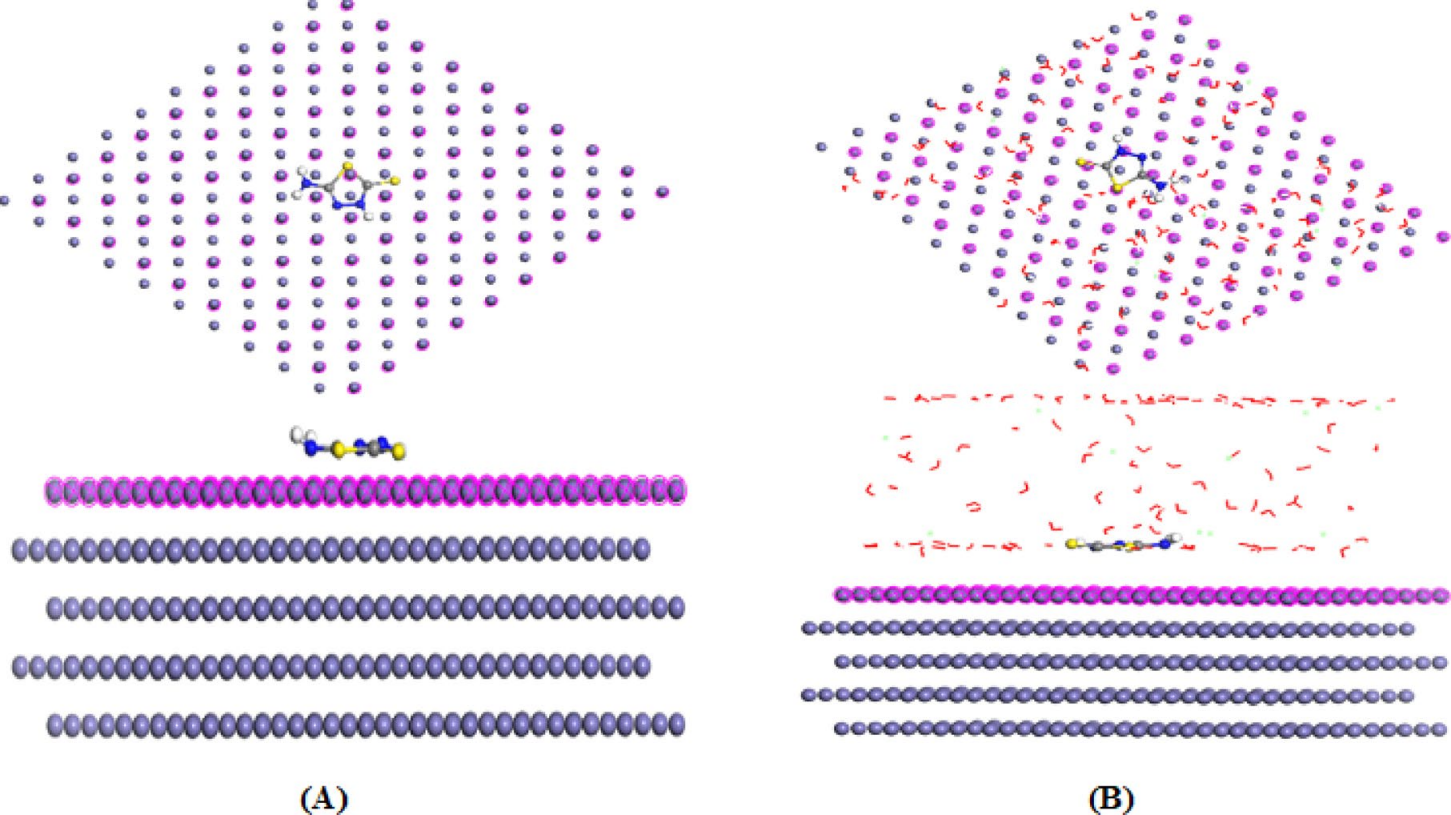
Equilibrium adsorption configuration of 5-ATT inhibitor, *thione* adsorbed on the Fe (110) calculated by MC simulation at room temperature: (**A**) gas phase; (**B**) solvent phase.

MC simulations were performed to elucidate the adsorption behavior of both *thione* and *thiol* tautomers of 5-ATT on the Fe (110) surface at the atomic level. The most stable adsorption configurations obtained in both gas and solvent phases are illustrated in Fig. 8 (thiol) and Fig. 9 (*thione*). In both environments, the inhibitor molecules adopt a nearly parallel orientation relative to the iron surface, thereby maximizing surface contact/coverage and facilitating strong interaction between the nitrogen and sulfur heteroatoms and surface Fe atoms^90–92^. The energetic parameters derived from MC simulations are summarized in Table 7. In the gas phase, the adsorption energies are − 81.244 kJ mol^−1^ for *thione* and − 78.869 kJ mol^−1^ for *thiol* tautomers. The slightly more negative adsorption energy of *thione* is consistent with DFT results, which indicated its lower ΔE and higher softness, favoring stronger donor–acceptor interactions under vacuum conditions^93^. The corresponding deformation energies (− 16.706 kJ mol^−1^ for *thione* and − 18.238 kJ mol^−1^ for *thiol*) indicate minimal structural distortion upon adsorption, reflecting good geometric compatibility with the Fe (110) surface^94^.

**Table 7:** The calculated energies by Monte Carlo simulation for *Thione* and *Thiol* tautomers of 5-ATT inhibitor in gas and solvent solution phases on Fe (110) surface.

Inh	Phase	E_T_ (KJ/mol)	E_ads_ (KJ/mol)	E_rig.ads_ (KJ/mol)	E_def._ (KJ/mol)	3D Atomistic	3D Atomistic H_2_O	3D Atomistic H_3_O^+^	3D Atomistic Cl^-^
Thione	Gas	− 101.428	− 81.244	− 64.538	− 16.706	− 81.244	-	-	-
	Solvent	− 2796.08	− 2873.16	− 2825.92	− 47.244	− 103.988	− 9.499	− 159.924	− 126.451
Thiol	Gas	− 102.395	− 78.869	− 60.632	− 18.238	− 78.869	-	-	-
	Solvent	− 2833.67	− 2906.38	− 2857.59	− 48.790	− 91.047	− 12.600	− 161.000	− 131.796

In contrast, a different trend emerges in the simulated acidic aqueous environment containing 100 H₂O, 5 H₃O⁺, and 10 Cl^−^ species^95^. The calculated adsorption energies become significantly more negative (− 2873.16 kJ mol^−1^ for *thione* and − 2906.38 kJ mol^−1^ for *thiol*), with the *thiol* tautomer exhibiting stronger overall adsorption. Although *thione* shows slightly stronger direct interaction with the bare Fe surface (3D atomistic interaction), *thiol* demonstrates more favorable interactions with surrounding H₂O, H₃O⁺, and Cl^−^ species, as evidenced by the more negative interaction energy components listed in Table 7. These findings indicates that in acidic aqueous media, adsorption is not governed solely by intrinsic electronic reactivity^96^. Instead, stabilization of the interfacial layer through hydrogen bonding, electrostatic interaction, and competitive displacement of corrosive species becomes a dominant factor. The presence of the –SH functional group in *thiol* tautomer enhances intermolecular stabilization and facilitates the formation of a compact protective film, consistent with previous simulation studies reporting strong sulfur-mediated metal–inhibitor coordination^96^. It is important to emphasize that the apparent difference between DFT and MC results arises from the different theoretical descriptions. While DFT reflects the intrinsic electronic reactivity of isolated molecules, MC simulations incorporate explicit interactions with the metal surface and surrounding environment. Under experimental acidic conditions, the thiol tautomer is expected to be the dominant adsorbing species due to its superior interfacial stabilization, despite the higher intrinsic reactivity of the *thione* form predicted by DFT. The outcomes of MC simulation indicate that the *thione* form is preferred under vacuum conditions owing to its greater electronic softness, whereas the *thiol* form becomes more stable in aqueous acidic media because of stronger interfacial stabilization. These findings bridge the DFT and MC outcomes and confirm that the tautomeric transformation of 5-ATT plays a key role in governing its corrosion inhibition mechanism across different environmental conditions.

### Proposed adsorption mechanism

**Fig. 10:**
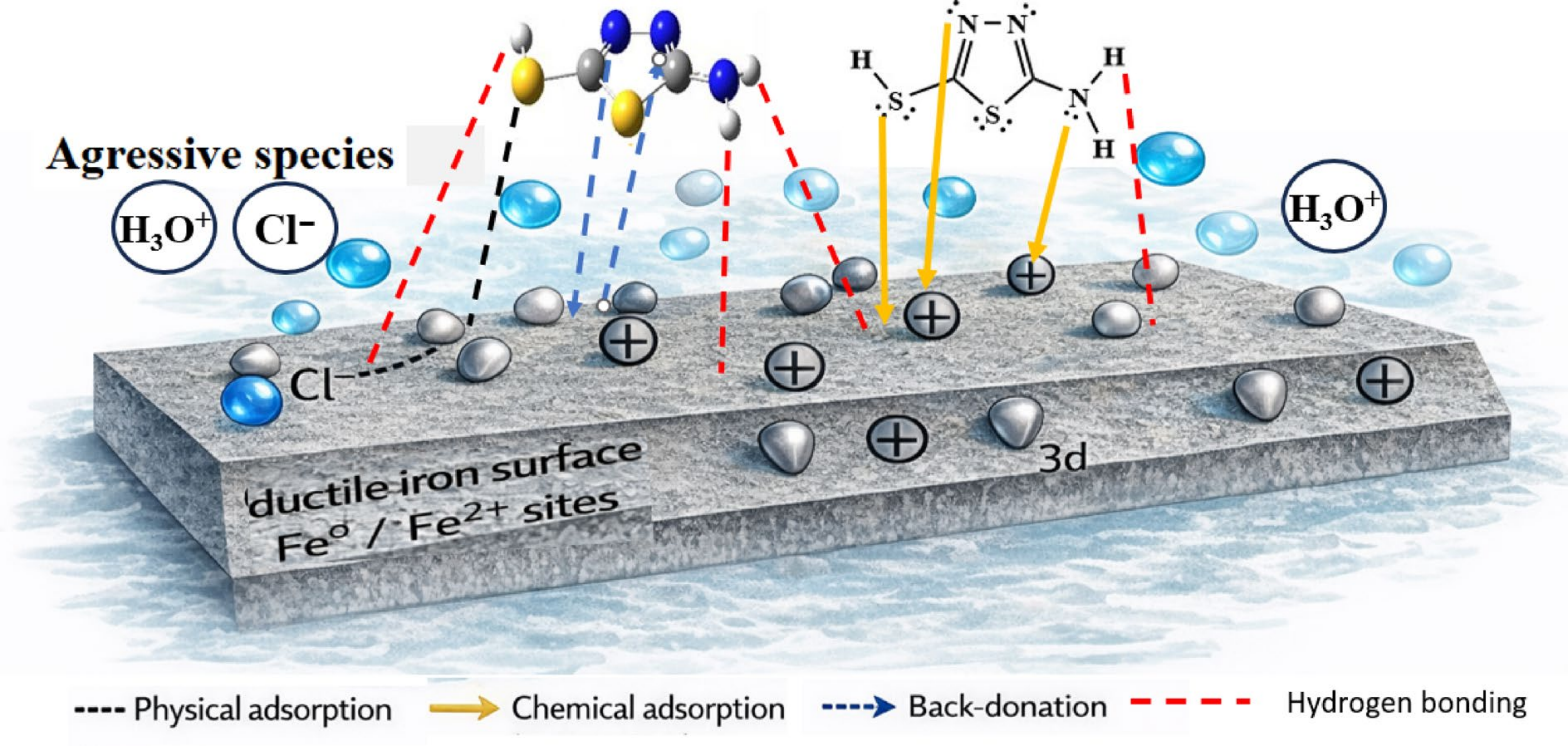
Schematic representation of the adsorption mechanism of 5-ATT inhibitor on Fe (110) surface in acidic medium.

Based on the combined experimental, electrochemical, surface, and theoretical findings, a comprehensive adsorption mechanism for 5-ATT on the Fe (110) surface can be proposed, as illustrated in Fig. 10**.** The adsorption process involves both physisorption and chemisorption mechanisms. Physisorption arises from electrostatic interactions between protonated inhibitor species and negatively charged sites on the metal surface, as well as interactions with adsorbed chloride ions. In parallel, chemisorption occurs through coordination between lone pair electrons of nitrogen and sulfur atoms and the vacant Fe 3d orbitals, forming stable donor–acceptor bonds. The thiol tautomer, in particular, plays a dominant role in aqueous environments due to the presence of the –SH group, which enhances adsorption via strong Fe–S interaction, hydrogen bonding with surrounding water molecules, and stabilization of the interfacial layer. Additionally, π-electrons of the thiadiazole ring contribute to surface interaction through π–d orbital overlap. The combined effect of these interactions leads to the formation of a compact and protective adsorbed film that effectively blocks active corrosion sites and suppresses both anodic and cathodic reactions.

## Conclusion

The corrosion inhibition effectiveness of 5-amino-1,3,4-thiadiazole-2-thiol (5-ATT) for ductile iron in 1.0 M HCl was assessed experimentally and complemented theoretically. 5-ATT functions as an effective mixed-type inhibitor, reducing both anodic and cathodic corrosion reactions, as consistently shown by the combined gravimetric, electrochemical, and surface investigations. The creation of a compact and protective adsorbed film at the metal–solution interface was indicated by the electrochemical data (polarization and EIS), which confirmed a large increase in polarization resistance accompanied by a decrease in double-layer capacitance.

SEM/EDX measurements, have demonstrated a noticeable change in surface morphology and a definite decrease in corrosion damage, provided additional evidence for this protective effect. Temkin, Flory–Huggins, and kinetic-thermodynamic isotherms all fit the heterogeneous, non-ideal adsorption behavior. The inhibitory process is carried out by a combination physisorption and chemisorption mechanism, according to thermodynamic factors. Theoretically, high adsorption and a stable parallel structure of 5-ATT on the Fe surface were confirmed by Monte Carlo simulations, while DFT calculations indicated nitrogen and sulfur atoms as the primary active centers responsible for surface interaction.

## Data Availability

All data generated or analyzed during this study are included in this published article.
